# Probabilistic Representation Differences between Decisions from Description and Decisions from Experience

**DOI:** 10.3390/jintelligence12090089

**Published:** 2024-09-20

**Authors:** Dandan Nie, Zhujing Hu, Debiao Zhu, Jianyong Yang

**Affiliations:** 1 School of Psychology, Jiangxi Normal University, Nanchang 330022, China; niedandan@jxnu.edu.cn (D.N.); zhudebiao@jxnu.edu.cn (D.Z.); 2 Mental Health Service Center, Huanghuai University, Zhumadian 463000, China; yangjianyong@huanghuai.edu.cn

**Keywords:** decision making, decision from description, decision from experience, description–experience gap, representation, probability, percentage, frequency

## Abstract

For mathematically identical risky decisions, different choices can be made depending on whether information about outcomes and their probabilities is learned by description or by experience, known as the description–experience gap. However, it is unclear whether different ways of obtaining information lead to different representation forms of probability, resulting in a description–experience gap. The current study investigates the representation formats of the alternative options’ probability for decisions from description and decisions from experience. The experiments measured the relative error of probability estimation in percentage and frequency forms for the two types of decisions in low and medium-to-large probability situations. The results demonstrate that for decisions from description in medium-to-large probability scenarios, the estimation error was lower in percentage form than in frequency form, with equally near-perfect estimates in small-probability scenarios. Nevertheless, in decisions from experience, the accuracy of estimation in percentage form was lower than in frequency form in both low and medium-to-large probability situations. This suggests that decision makers in decisions from description tend to represent the probability information in percentage form. However, in decisions from experience, they tend to represent probability in frequency form. The utilization of different formats for probability representation is one of the factors that contribute to the description–experience gap.

## 1. Introduction

### 1.1. Description–Experience Gap

In the real world, individuals can make decisions based on descriptive information that provides exact outcomes and their corresponding probabilities. Additionally, people can make appropriate decisions based on their experience. However, the way people receive information has a significant impact on their decisions. For example, parents and doctors have different attitudes when deciding whether to immunize one’s child. Parents make decisions based on descriptive information, the direct presentation of an adverse reaction’s outcome and probability, as conveyed by a doctor or a website. They may *overestimate* the consequences of these small probability adverse events leading to resistance to vaccination. While doctors often consider their own previous vaccination experience and make decisions based on that experience, they may *underestimate* the small probability of adverse events and incentives to vaccination.

In a study conducted by Barron and Erev (2003), it was first discovered that individuals exhibit distinct behavioral patterns in the context of traditional description-based decisions and feedback-based decisions. This finding has prompted further investigation. Building upon this, Hertwig et al. (2004) formalized the study of decisions from description (DFD) and decisions from experience (DFE). In the traditional DFD paradigm (also known as description-based decisions or descriptive decisions), individuals make a series of decisions among options that explicitly identify possible monetary outcomes and their probabilities in a numerical format. In the DFD paradigm, decision makers explicitly know the outcomes of options and the corresponding probabilities, and they can make decisions based on this information. In contrast, in DFE paradigms (also known as experience-based decisions or empirical decisions), decision makers must also select a preferred option. However, in this case, decision makers do not know the magnitudes and probabilities of the outcomes and can only infer them through sequential sampling and feedback (Hertwig et al. 2004; Wulff et al. 2018). 

The past two decades have seen a proliferation of research exploring the distinctions between decisions from description and decisions from experience (Park et al. 2021). The experimental evidence consistently shows that systematically different choices are made between DFD and DFE, even if the experienced or described information conveys mathematically equivalent information (Aydogan and Gao 2020; Dai et al. 2019; Hertwig et al. 2004, 2019; Hertwig and Wulff 2022; Pindard-Lejarraga and Lejarraga 2024). This phenomenon is called the description–experience gap (D-E gap) (Hertwig and Erev 2009). The discrepancy in subjects’ weights for low-probability events is the most typical discrepancy observed between the two types of decisions. When making decisions based on explicitly described information, decision makers behave as if they overestimate or overweight rare events and underweight moderate-to-high probability events, as described by cumulative prospect theory (Kahneman and Tversky 1979). The nonlinear weighting of probabilities results in a fourfold-pattern in people’s risk attitudes (Kahneman and Tversky 1979; Tversky and Kahneman 1992). Decision makers tend to exhibit risk-seeking behavior in scenarios with a low probability of gain and risk-averse behavior in scenarios with a medium or high probability of gain. Conversely, they tend to exhibit risk-averse behavior in scenarios with a low probability of loss and risk-seeking behavior in scenarios with a medium to high probability of loss. However, when decisions are made based on experience, decision makers behave as if they underestimate or underweight rare events and overweight moderate-to-high probability events (Camilleri and Newell 2011; Hertwig et al. 2004; Lejarraga and Gonzalez 2011; Wulff et al. 2018). And their risk attitudes exhibit a reversed fourfold pattern. 

### 1.2. Mechanisms of the Description–Experience Gap

The long history of discussion about the mechanisms of the description–experience gap can be summarized in two main aspects: sampling error and cognitive factors. The sampling error is ubiquitous in the experience-based decision paradigms with optional stopping. Due to the opportunity cost, time cost, and capacity limitations of working memory (Hoffart et al. 2019), people tend to rely on small samples of information to make decisions, sampling approximately 11 to 19 times for each question (Hau et al. 2010), i.e., 5 to 10 times for each option. The frequency of small-probability events exhibits a skewed binomial distribution when only very small samples are taken. This leads to sampling error in empirical decision making. In other words, the actual empirical number of small-probability events is not equal to the theoretical number of probabilities multiplied by the number of sampling occasions (Hertwig et al. 2004). 

However, many studies have demonstrated the robustness of the D-E gap, which remains stable after excluding the sampling error (Hoffart et al. 2021; Ungemach et al. 2009; Wulff et al. 2018). Thus, the D-E gap is not an artifact of statistical error but rather a robust systematic difference. It could be explained in terms of the information processing factors (Camilleri and Newell 2013), i.e., memory, representation, and evaluation. Each section can lead to discrepancies between DFD and DFE. A functional magnetic resonance imaging study demonstrated that individuals activate distinct neural networks in the brain when making descriptive and empirical decisions (FitzGerald et al. 2010).

### 1.3. Frequency Representation and Probability Representation

At the cognitive level, the information an individual acquires from both description and experience must be represented in the mind. This part of the information processing may also result in the D-E gap occurring. The main formats for characterizing numerical information are percentage or probability and frequency. In human evolutionary processes, the concept of percentage or probability emerged relatively late. Prior to this, humans were unable to use probability or percentage to characterize numbers. So how did humans characterize numbers at that time? Scientists have proposed that the initial method of characterizing numbers may have been natural frequencies in the evolutionary process (Gigerenzer and Hoffrage 1995). Natural frequency is a way of characterizing numbers obtained through natural sampling.

The format of information can influence judgment and decision making. Research in the field of probabilistic reasoning has demonstrated similar results. For instance, replacing the probabilistic format with the frequency format for information representation improved people’s performance in solving Bayesian inference problems (Gigerenzer and Hoffrage 1995). How probability is represented can affect the decisions made in different contexts (Hau et al. 2010).

With regard to cognitive processes, the representation of information in a frequency format appears to simplify calculations, reduce the burden of attention, make it easy to ignore the base rate, and facilitate the validation of the posterior distribution. The frequency representation format involves more information regarding individual experience and natural sampling than the probability representation format. It is noteworthy that mathematically equivalent information representations may not be equivalent in cognitive algorithms. For instance, researchers examined the performance of subjects in Bayesian inference tasks presented in both probability and frequency formats. The results demonstrated that only 8% of the subjects reasoned correctly when Bayesian inference was conducted in the probability format, whereas 46% of the subjects reasoned correctly when Bayesian inference was conducted in the frequency format (Gigerenzer and Hoffrage 1995). Additionally, it was demonstrated that frequency is the most suitable format for achieving Bayesian inference among the three forms of probabilistic representation, namely percentage, frequency, and probabilistic forms (Yang and Hu 2007). 

In the descriptive and empirical decision-making paradigms, risk information is conveyed to decision makers in different forms. In descriptive decision making, probabilities are usually conveyed in written form as percentage information (e.g., an 80% probability of receiving USD 4). In empirical decision making, however, the information is obtained empirically through sequential sampling, must be interpreted by the decision maker, and may be influenced by perceptions (e.g., this option gives me a good reward 8 times out of 10) (Cubitt et al. 2022). Hertwig and Erev (2009) link the analogy of findings in probabilistic learning and reasoning studies to empirical decision making. They suggest that the D-E gap may be due to the fact that information presented in different formats triggers different cognitive mechanisms or cognitive algorithms.

Arithmetic equivalence does not mean psychological equivalence. The manner in which information is represented can significantly influence decision-making processes (Hau et al. 2008, 2010). For instance, “80%” is the same as “8 out of 10” in math. Nevertheless, the interpretation of these two types of information may diverge in mental calculations, which could potentially impact the outcome of a decision.

In traditional descriptive decision-making tasks, the probability of an outcome is presented in terms of the probability or percentage of a single event, whereas, in empirical decision making, one needs to learn about a sequence of events and estimate the likelihood of a decision option through a sequential information sampling process. This process involves the potential that individuals may be more adept at processing the frequency (as opposed to the probability) of events and have the tendency to activate frequency thinking more preferentially. The divergence in information representation may prompt individuals to utilize different decision rules, thereby engendering a D-E gap (Camilleri and Newell 2013).

For decisions from description, compensatory decision rules, as represented by prospect theory, predict decision behavior well, suggesting a “weighted summation” process in which individuals make judgments based on comparing the value of each decision option (Kahneman and Tversky 1979). To calculate the value, individuals must integrate the outcomes’ probability and magnitude, so they must accurately represent the probability. In contrast, for decisions from experience, individuals learn decision-making information through sampling and feedback. In a sampling paradigm, where the goal is to decide which option is “better,” the decision maker may prefer a satisficing heuristic and attempt to make a decision with minimal computational effort (Camilleri and Newell 2009). Consequently, in the context of representations, the fundamental requirement for this task is to identify the optimal option, irrespective of the extent to which it is superior or the probability of each outcome. A cognitive model fitting study (Hoffart et al. 2021) suggests that most participants’ risk representations can be summarized in cognitively simpler, relative frequency-based cognitive models. Consequently, for empirical decisions, it is not necessary to make propositional statements regarding the probabilities of all outcomes, as is carried out in descriptive decisions.

Previous research (Camilleri and Newell 2009) employed verbal and nonverbal assessment probes to investigate subjects’ mental representation of outcome distributions in both DFD and DFE. The verbal probe requested subjects to fill in the sentence, “__% of cards were worth __ points”. The nonverbal probe consisted of a series of small squares forming a large grid; the subjects were asked to adjust the density of the grid up and down with keystrokes to match their impressions of the probability of each option. The results indicated that the subjects demonstrated more accurate probability judgment in DFD using the verbal probability probe, and more accurately in DFE using the nonverbal density probe. Individuals are more likely to engage in non-probabilistic thinking when probabilistic cues are relatively unclear (Rottenstreich and Kivetz 2006). There are no explicit probabilistic cues in DFE compared to DFD. Furthermore, probabilistic cues are less salient in DFE than in DFD. Consequently, subjects may be more inclined to characterize the magnitude of the risk in the form of frequencies in decisions from experience. It is possible that subjects may only recall the frequency of a specific outcome or the approximate number of times it is presented rather than the probabilistic representation itself. 

The findings of a recent study also support this possibility. The research demonstrated that part of the effect of experience reflects the belief that the environment is dynamic, and the optimal strategy for decision making is to discover patterns or regularities rather than estimate probabilities (Plonsky et al. 2015). If people search for patterns in DFE, they are less likely to estimate proportion but still might recall the frequency of the rare outcomes. People can sample from their memories of past experiences with each option and choose the option with the higher sample average. The instance-based learning theory (IBLT) has a similar point of view (Gonzalez et al. 2003). The model assumes that decision making is based on the storage and retrieval of instances of past decisions. An instance includes decision outcomes and environment variables. When facing a decision problem, individuals need to recall instances from memory and make decisions based on these instances. These observations provide a more natural motivation for the current hypothesis that agents may be better at retrieving the frequency of events than at estimating their probability or percentage in DFE. 

Based on the aforementioned findings, we hypothesized that there are clear probabilistic cues in DFD, individuals tend to think in explicit forms of probabilities, and the representation of data is in the form of percentages. In contrast, the probabilistic cues in DFE are less explicit than those in DFD. Individuals tend to think in a non-probabilistic manner, and the representation of information is in frequencies. The different formats of representation are one of the factors contributing to the D-E gap.

### 1.4. The Current Study

Despite the growing number of studies on the cognitive mechanisms underlying the description–experience gaps, the question of how representation plays a role in the D-E gap remains relatively unexplored. The current study aims to further examine the mechanisms underlying the description–experience gap at the information representation level. That is, we explore whether there is a difference in the manner in which probabilistic information is represented by the two types of decisions. Therefore, we conducted two experiments to explore whether DFD and DFE differ in the form of representation of the magnitude of the risk (i.e., probability) in small and medium-to-large probability situations. In both experiments, the subjects received information and made decisions in either a descriptive or empirical manner. They were subsequently asked to estimate the magnitude of the risk associated with an option in the form of frequency or probability. The estimation error in the two forms was then compared. It was hypothesized that if the subjects’ representation of the risk information is in percentage form, the estimation error of the frequency form would be lower than that of the percentage form. Conversely, if the subjects’ representation of the risk information is in the frequency form, the estimation error of the frequency form would be lower than that of the percentage form. Based on the results of previous studies, this study predicted that (1) when making decisions based on descriptions, the subjects would demonstrate a lower or equally high estimation error of risk in the percentage form, and (2) when making decisions based on experience, the subjects would demonstrate a lower estimation error of risk in the frequency form. It was expected that the respondents in DFE would prefer the frequency form of representation relative to DFD.

## 2. Experiment 1

Experiment 1 investigated the participants’ representation of risk in both DFD and DFE in a small-probability situation. Based on previous research, the small-probability setting was defined as 0.25 and below (Hertwig et al. 2004).

### 2.1. Method 

#### 2.1.1. Participants and Design

Experiment 1 employed a mixed design of 2 methods (estimate approach: percentage, frequency) × 2 paradigms (decision type: decision from experience (DFE), decision from description (DFD)). The decision type was a between-subjects variable, while the estimate approach was a within-subjects variable. The primary motivation for employing mixed design was to avoid the potential for the different cognitive processes associated with the two decisions to exert an influence on one another. Distinct cognitive processes underlying DFE and DFE have been demonstrated (Glockner et al. 2012). Consequently, the majority of studies that compare descriptive and empirical decision-making processes have subjects making either a descriptive decision or an empirical decision, respectively (Barron and Erev 2003; Glockner et al. 2012; Hau et al. 2008, 2010; Hertwig et al. 2004; Kopsacheilis 2018; Ungemach et al. 2009). Additionally, other studies employed a within-subjects design, but the two sessions (DFD, DFE) were separated by at least one week (Kellen et al. 2016). Based on previous studies, the current experiment randomly assigned half of the participants to the DFD condition and half to the DFE condition.

The dependent variable was a measure of estimation error: the absolute value of the difference between the true value (t) of the percentage or frequency and the estimated value (x) of the rare event divided by the true value. This was expressed as |t − x|/t. Low error means high accuracy. For example, in the case of “a 0.2 probability of getting CNY 5”, the true value of the percentage is 20%. Consequently, if the subject inputs “10”%, the estimation error will be calculated as 0.5 (|20 − 10|/20).

A priori power analysis using G*power 3.1 (Faul et al. 2009) was conducted to estimate the necessary sample size. We set the effect size as *f* = 0.25, *α* = 0.05, power (1 − *β*) = 0.95. The results indicated that a minimum of 52 participants was required. A total of 60 college students (27 males; age: 21.97 ± 1.67 years) were recruited and randomly assigned to the empirical and descriptive decision-making groups. All subjects were free of any history of neurological or psychiatric disorders, and they had normal or corrected-to-normal vision. Prior to the experiment, all participants provided informed written consent. Each participant was paid for participating in the experiment, including a base fee of 10 Yuan (CNY) and earnings based on their actual performance.

#### 2.1.2. Materials

The formal experiment consisted of six pairs of decision problems, each with one risky option with a small probability of winning and another certain option (see Table A1). For example, “Option A: CNY 10 with 0.2, CNY 0 otherwise; Option B: CNY 2 for sure”. In order to avoid fatigue effects (especially under DFE conditions) and ensure sufficient testing of the subjects’ representations, only six formal trials were conducted for each condition based on previous research. Particularly under DFE conditions, the participants were required to explore 8 or 10 times per option (16 or 20 times per decision problem) to learn the distribution of potential outcomes of options. An excessive number of trials will inevitably result in fatigue. We balanced the experimental efficiency and subject fatigue, and the number of formal trials for the present experiments was ultimately set to approximate that of previous related studies, such as the related research of Camilleri and Newell (2009, 2011), Hertwig et al. (2004), Hau et al. (2008), Hoffart et al. (2021), Klingebiel and Zhu (2022), which set up about 6–10 gambling pairs.

#### 2.1.3. Task and Procedure

The subjects were randomly assigned to either the descriptive or the empirical condition. They were provided with identical information regarding the potential outcome and probability of a decision problem in either a descriptive or empirical way. Then, they made a choice based on the information provided. Finally, the respondents were asked to report the likelihood of the risky option winning money in percentage and frequency form. Before the formal experiment, the participants were asked to read the instructions and complete two exercises to ensure a comprehensive understanding of the experimental procedure. Any questions that arose during the practice sessions were encouraged to be reported to the experimenter, who provided answers and additional practice opportunities.

In the DFD condition, the subjects were directly provided with complete decision information about the potential outcomes of options and their corresponding probabilities on the computer screen. In the DFE condition, the participants were presented with two buttons representing the two decision options. They were instructed to sample each option to learn the options’ underlying outcomes and corresponding likelihoods before proceeding to the decision phase. The number of samples was set to 8 or 10 per option, and 16 or 20 per problem. After each sampling, the participants received feedback, with the selected, not the foregone, outcome displayed on the screen for the corresponding button. It is worth noting that the present experiment eliminated sampling bias by ensuring that the empirical frequencies of outcomes were precisely equal to the theoretical frequencies (i.e., the probability multiplied by the number of samples). For instance, the decision problem with a 0.2 probability of winning CNY 10 and a 0.8 probability of winning nothing would be implemented precisely 2 times with the CNY 10 outcome and 8 times with the CNY 0 outcome out of 10 samples, in random order. And if the probability was 0.25, the participants would encounter the event 2 times out of 8 samples.

Previous research typically ensured a representative sample by ensuring that the empirical probabilities were precisely equal to the descriptive probabilities (Ungemach et al. 2009), but did not fully consider the sample size. However, individuals rely on small sample sizes in natural sampling (Plonsky et al. 2015), approximately 5–10 per option in the free sampling paradigm (Hau et al. 2010), close to the capacity of working memory, which is 7 ± 2 units. The current experiments employed a small sampling approach, which is more ecologically valid. Another important reason for small sampling is that increasing the number of samples would lead to internal sampling bias associated with memory loads (Camilleri and Newell 2011). Given these considerations, the experiment was designed with a relatively limited sample size. The position of the two gambles on the screen (i.e., left or right) was counterbalanced.

**Figure 1:**
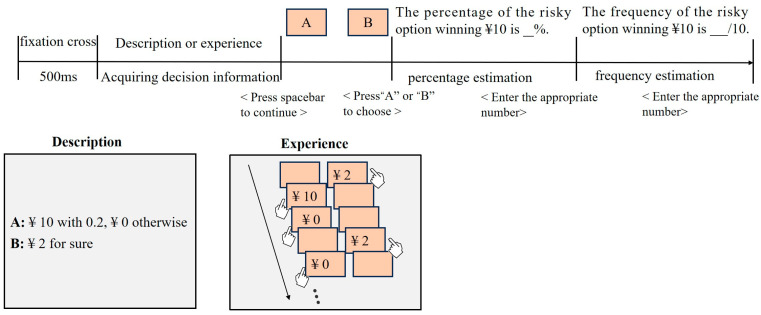
Procedure of the experiment. Note that the sequence of percentage estimates and frequency estimates was counterbalanced within the subjects.

After the participants received information about a given risk item, either empirically or descriptively, and made a decision, they proceeded to estimate and input the likelihood of winning for the risky option into the computer in the form of percentage or frequency. The percentage and frequency approaches are expressed respectively as “the percentage of the risky option winning CNY 10 is %” and “the frequency of the risky option winning CNY 10 is /10”. The order of presenting the decision problems was randomized, and the order of the estimation methods (percentage estimation first or frequency estimation first) was balanced. The formal experimental phase required, on average, approximately 6 min for the DFD condition and approximately 13 min for the DFE condition. Figure 1 illustrates the procedure of the experiment.

### 2.2. Results

Prior to conducting the formal analysis, we performed a normality test on the data for each group and determined that the data did not adhere to a normal distribution (*p*-value of the Shapiro–Wilk test below 0.05). Consequently, we opted to forego the parametric test and instead employed a nonparametric test. 

The results of the Wilcoxon signed ranks test revealed that, for DFD, the estimation error of percentage estimation (*Md* = 0.00 (0.00~0.16)) was not statistically different from that of frequency estimation (*Md* = 0.00 (0.00~0.08)), *Z* = −0.20, *p* = 0.844. For DFE, the estimation error of percentage estimation (*Md* = 0.07 (0.03~0.23)) was significantly higher than that of frequency estimation (*Md* = 0.00 (0.00~0.08)), *Z* = −2.23, *p* = 0.026. 

**Figure 2:**
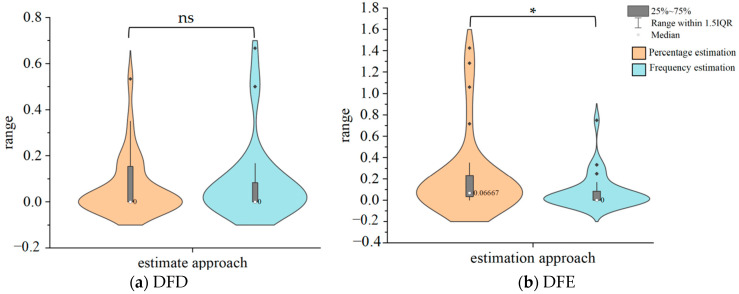
Estimation error of percentage estimation and frequency estimation in DFD (**a**) and DFE (**b**) (low-probability situation). The width of the graph represents the density of the data distribution. * means *p* < .05, ns indicates not significant. The interquartile range (IQR) is a statistical measure of dispersion. It is calculated by subtracting the third quartile from the first quartile. The black dots represent the extremes of the distribution.

Figure 2 presents violin plots of the estimation error for percentage estimation and frequency estimation in DFD and DFE.

As shown in Figure 2, in the low-probability situation, individuals in the DFD condition demonstrated high accuracy (low estimation error) when estimating risk in either a probability or frequency estimation manner. In the DFE condition, the estimation error of individuals estimating risk in a frequency estimation manner was significantly lower than the accuracy of individuals estimating risk in a percentage estimation manner. This suggests that individuals’ representation of risk in the DFE condition was predominantly in terms of frequency. In the DFD condition, individuals may have accurately represented the probabilistic information and processed it at a deep level to accurately convert percentages to frequencies. As can be observed, the participants in DFD conditions exhibited a nearly perfect estimation of risk. Experiment 2 investigated different ways of estimating probabilities in medium- to large-probability scenarios. The procedure for Experiment 2 was identical to that of Experiment 1. 

## 3. Experiment 2

Experiment 1 investigated the participants’ representation of risk probabilities in a situation where risky options had a low probability of winning. Previous research has demonstrated that people behave differently in low-probability situations and medium- to large-probability situations. However, it remains unclear whether there is a difference in people’s representation of risk probabilities between the low and the medium to large probability. To shed light on the form of the mental representation of probability regarding DFD and DFE across all probability conditions, we conducted Experiment 2 to investigate the participants’ representation of risk in both DFD and DFE in the medium- to large-probability situation. 

### 3.1. Method 

#### 3.1.1. Participants and Design

Experiment 2 employed a mixed design of 2 (estimate approach: percentage, frequency) × 2 (decision type: decision from experience (DFE), decision from description (DFD)). The decision type was a between-subjects variable, while the estimate approach was a within-subjects variable. The dependent variable was a measure of estimation error: the absolute value of the difference between the true value (t) of the percentage or frequency and the estimated value (x) of the common event in the risky option divided by the true value. This was expressed as (|t − x|)/t. For example, in the case of “a 0.8 probability of getting CNY 5”, the true value of the percentage was 80%. Consequently, if the subject input “40%”, the estimation error was calculated as 0.5 (|80 − 40|/80).

The majority of subjects in Experiment 1 proceeded with Experiment 2 after a 5 min rest, except for 2 subjects who were unable to continue due to scheduling conflicts. A total of 60 college students (27 males; age: 21.87 ± 1.60 years) were randomly assigned to empirical and descriptive decision-making groups. All subjects were free of any history of neurological or psychiatric disorders, and they had normal or corrected-to-normal vision. Prior to the experiment, all participants provided informed written consent. Each participant was paid for participating in the experiment, including a base fee of 10 Yuan (CNY) and earnings based on their actual performance.

#### 3.1.2. Materials

The formal experiment consisted of eight pairs of decision problems, each with one risky option with a medium to large probability of winning and another certain option (see Table A2). 

#### 3.1.3. Task and Procedure

The procedure for Experiment 2 was the same as for Experiment 1, except that the material used in Experiment 2 was set in a scenario with a medium to large (>0.25) probability of benefit (see Table A2). The formal experimental phase required, on average, approximately 10 min for the DFD condition and approximately 20 min for the DFE condition. 

### 3.2. Results

Prior to conducting the formal analysis, we performed a normality test on the data for each group and determined that the data did not adhere to a normal distribution (*p*-value of the Shapiro–Wilk test below 0.05). Consequently, we opted to forego the parametric test and instead employed a nonparametric test. 

The results of the Wilcoxon signed ranks test revealed that for DFD, the estimation error of percentage estimation (*Md* = 0.03 (0.02~0.05)) was significantly lower than that using frequency estimation (*Md* = 0.04 (0.02~0.07)), *Z* = −2.45, *p* = 0.014. For DFE, the estimation error of percentage estimation (*Md* = 0.05 (0.03~0.17)) was significantly higher than that of frequency estimation (*Md* = 0.03 (0.00~0.10)), *Z* = −4.10, *p* < 0.001. 

**Figure 3:**
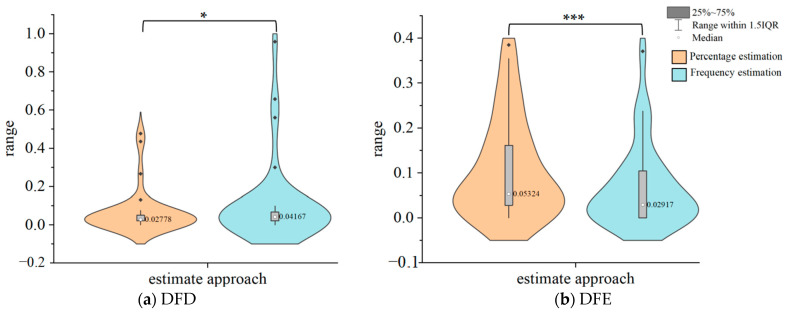
Estimation error of percentage estimation and frequency estimation in DFD (**a**) and DFE (**b**) (medium- to large-probability situation). The width of the graph represents the density of the data distribution. * means *p* < .05, *** means *p* < .001. The interquartile range (IQR) is a statistical measure of dispersion. It is calculated by subtracting the third quartile from the first quartile. The black dots represent the extremes of the distribution.

Figure 3 illustrates the violin plots of the estimation error for percentage estimation and frequency estimation in DFD and DFE.

The results of Experiment 2 are similar to those of Experiment 1. For the medium- to large-(>0.25) probability situation, individuals in the DFD condition demonstrated higher accuracy (low estimation error) in estimating risk using percentage than that using frequency estimation. In the DFE condition, the accuracy of the individuals estimating risk in a frequency estimation manner was significantly higher than that of the individuals estimating risk in a percentage estimation manner. This suggests that individuals’ representation of risk in the empirical condition was predominantly in terms of frequency, however, their representation of risk in the descriptive condition was predominantly in terms of percentage. Perhaps the tendency of underweight events with medium to large probabilities (Kahneman and Tversky 1979), makes them less critical to the decision maker or less salient in memory than small-probability events. As a consequence, recall of information related to these common events was more likely to be inaccurate, resulting in a reduction in estimation accuracy compared to that observed in Experiment 1. 

## 4. Discussion

Our goal was to investigate whether the representation of information in a decision from description and experience is different. The results indicate that there are different kinds of information representation in DFD and DFE. The manner of representation is a contributing factor to the D-E gap. The current experiments have shown that the participants in DFD exhibited a nearly perfect estimation of the winning probability of risky options in a small-probability scenario (Experiment 1), either in percentage or frequency form. In the small-probability scenario (Experiment 2), the error in frequency estimation was smaller than the error in percentage estimation. It is possible that the participants in DFD had a precise representation of probability; they tended to prefer percentage representation, where the information was so deeply processed it could be converted smoothly into percentages and frequencies. For DFE, the participants exhibited higher estimation accuracy for the frequency form than for the percentage form. This suggests that the representation form of probability information in DFE tends to be in frequency form, and that the information may not be deeply processed. Therefore, more cognitive resources are required when the frequency is converted into a percentage, leading to a decrease in accuracy when estimated in a percentage format. 

To rule out the possibility that people would make mistakes because division (in the form of percentage estimates) is harder, the current experiments were designed with simple risk probabilities (e.g., 0.1, 0.2) and simple sampling size (e.g., 10), etc. so that the subjects were able to calculate the percentages. i.e., 10%, 20%, without additional effort. For two-digit probabilities with minimal difficulty, e.g., 0.25, a pre-rehearsal was conducted in the practice phase of the experiment, where 2 out of 8 samplings were experienced, and the participants were encouraged to report an outcome of 25%. With these experimental settings, the additional effect of computational factors was eliminated. The decrease in the subjects’ percentage estimation accuracy relative to their frequency estimation accuracy in the DFE condition may be related to *base rate neglect* (Kahneman and Tversky 1973) under heuristic processing. This phenomenon can be defined as the tendency for individuals to rely on specific information and neglect general information (i.e., the denominator) when making subjective judgments. 

In decisions from experience, individuals seem to be more adept at initiating frequency thinking and not good at utilizing probabilistic information precisely. This phenomenon is related to the evolutionary process of the development of human thinking. In the evolutionary process of human ancestors, numerical information was not initially characterized in probability and percentage format. Instead, it was characterized in the form of natural frequencies. Frequency is a form of numerical representation obtained through natural sampling, which is the same way people acquire information in an empirical decision-making environment. 

The representation of DFE is frequently in the form of frequencies, as compared to DFD. This aligns with the findings of (Camilleri and Newell 2009); the goal of empirical decision making is to identify which option is “better” instead of performing a precise value calculation based on percentages or probabilities to determine the degree of advantage. The decision maker can use a satisficing heuristic and attempt to make a decision with the lowest computational effort. It is possible that probabilistic information is represented in an imprecise manner in DFE. This imprecise use of probabilities is consistent with the heuristic view and may further lead to a different prioritization of DFD and DFE with regard to analytical and heuristic strategies.

In the dual process theory (Evans 2003) of reasoning and decision making, analytic strategies, which are represented by valuation prioritization, and heuristic strategies, which are represented by pairwise comparisons, lie at opposite ends of an antithetical continuum depending on the degree of use of quantitative tools, such as probability. Analytical strategies are characterized by a weighted summation rule that assigns mental weights to the product of the probability function and the magnitude of the outcome. As a result, analytical strategies involve the precise representation and operation of probabilistic information. In contrast, heuristics assume that humans have limited rationality and limited computational capacity and that individuals are unable to integrate probabilities and outcomes fully. This may result in individuals comparing between some particular dimensions of options rather than between the value of options (Liu et al. 2022; Rao et al. 2013; Su et al. 2013). As a result, individuals may be inclined to ignore probabilities, leading to imprecise representation and the exploitation of probabilities when using heuristic strategies. 

There have been some clues that subjects prefer heuristic strategies for empirical decisions and analytic strategies for descriptive decisions. When individuals were exposed to a description and experience simultaneously, the influence of experience dominated the influence of description (Weiss-Cohen et al. 2016). Participants preferred empirical information over descriptive information when given the choice of using either descriptive or empirical information for decision making (Lejarraga 2010). The prevalence of empirical information in this context aligns with the dual processing model of reasoning and decision making; when the heuristic system and the rational analysis system conflict, they compete with each other, and the heuristic system usually prevails. 

Different theoretical models have different assumptions about how people represent and exploit probability. Instance-based learning models do not directly assess probabilities (Gonzalez et al. 2011). In contrast, such models suggest that individuals either develop expectations regarding the mean return of the options or sample from memories of previous encounters to estimate the relative frequency of potential prospects. However, the reinforcement learning CPT hybrid model (Haines et al. 2018) hypothesizes that individuals learn the outcomes’ probabilities directly before integrating the outcome sizes and their corresponding probabilities. 

Different theoretical models make various assumptions about how people learn and exploit probability in their environments, which involves different methods of information processing. Future research can further test the applicability of these theories to the information processing process of decision making. Specifically, decision making based on experience relies more on memory and learning processes than descriptive decision making. Focusing on two key ways in which people acquire information, description and experience, and analyzing the decision-making process for the two corresponding decisions has contributed to a more nuanced understanding of how people perceive and respond to risk in different decision-making contexts. This has led to the accumulation of valuable evidence for predicting human responses to risk (Hertwig and Wulff 2022). Studying how individuals represent probabilities in description and sampling (experience) situations can help explore the most appropriate ways for individuals to comprehend mathematical information in different learning situations. Adopting optimal learning styles can make the learning process more efficient.

Our experiments employed a mixed design, with half of the subjects completing DFD and the remaining half completing DFE. Further research could include additional trials but with complementary conditions. For example, one group could complete DFD, and then be required to complete DFE again, while the other group would conduct the opposite. This would allow researchers to analyze the initial trials between groups and also to analyze the initial and subsequent trials within groups, testing whether this makes a difference. In this way the effects of DFD and DFE could be analyzed. 

## 5. Conclusions

Overall, the current study found that different ways of acquiring information lead to different forms of representation of information. When making decisions from description, individuals are relatively better at percentage representation than frequency representation. And they may accurately represent the probabilistic information at a deeply processed percentage level, so one could accurately convert percentages to frequencies in the DFD condition. However, when making decisions from experience, individuals tend to favor a frequency representation. One of the factors that contribute to the D-E gap is the different formats of risky representation. 

## Figures and Tables

**Figure A1:**
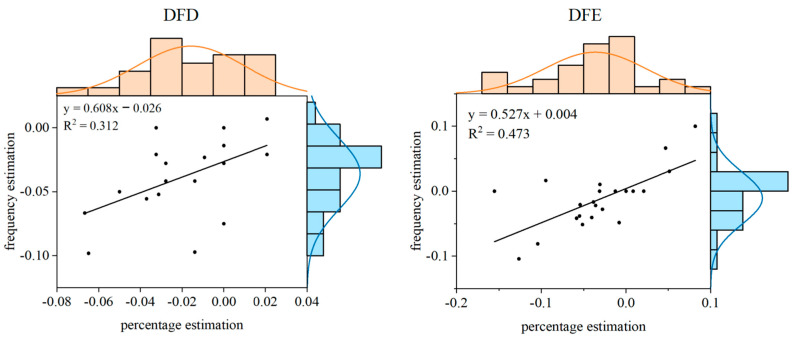
Relationship between percentage estimation and frequency estimation in DFD and DFE (medium- to large-probability situation). Black dots are associated with the scattering of data. The solid line depicts the least-square regression lines describing the relationship between the estimation error of percentage estimation and frequency estimation. The orange and blue bars represent histograms of the x-axis data and y-axis data, respectively. The curves represent the respective normal fit curves.

## Data Availability

The datasets generated and analyzed during the current study are available from the corresponding author upon reasonable request.

## Appendix A

As this study was not concerned with the direction of error, only the magnitude of error was of interest. Therefore, in Experiments 1 and 2, the absolute value of the estimation error was calculated. The following section presents an analysis of the relative values of errors (with directions) for those interested in reading them.

In addition, we analyzed the reasons for the non-normality of the data. One potential explanation for this skewing is the use of absolute values in the calculation of estimated errors (|t − x|/t), which resulted in the data being skewed because they were all greater than zero. However, for Experiment 1, since the estimation was performed in a small-probability scenario, events that occurred once or twice had high memory salience, enabling most subjects to accurately judge the frequency or percentage. Consequently, there were more values of 0, and the distribution remained skewed even without the use of absolute error (substituted for (t − x)/t). The results are similar to those of the main part of Experiment 1.

In Experiment 2 (medium- to large-probability scenario), the distribution was normal when the absolute error was dropped (substituted for (t − x)/t). The data were found to be normally distributed. That is, there were overestimated and underestimated percentages and frequencies. We analyzed this and plotted a linear model, with the error of percentage estimation on the x-axis and the error of frequency estimation on the y-axis (Figure A1).

The results of the normal distribution test (Shapiro–Wilk test) indicate that the data are normally distributed. For percentage estimation in DFD, *W* = 0.94, *p* = 0.115; for frequency estimation in DFD, *W* = 0.94, *p* = 0.138. For percentage estimation in DFE, *W* = 0.95, *p* = 0.268; for frequency estimation in DFE, *W* = 0.92, *p* = 0.050.

The results of ANOVA indicate that the main effect of the estimate approach and the decision type was not significant, but the interaction between the decision type and the estimate approach was significant *F*(1,51) = 18.50, *p* < 0.001, *η_p_*^2^ = 0.27.

Simple effects analyses revealed similar results as when calculating the absolute error. For DFD, the estimation error (*M* = −0.015, *SE* = 0.08) of percentage estimation was statistically different from that of frequency estimation (*M* = −0.037, *SE* = 0.07), *F*(1,51) = 11.060, *p* = 0.002, *η_p_*^2^ = 0.178. For DFE, the estimation error of percentage estimation (*M* = −0.027, *SE* = 0.008) was also significantly different from that of frequency estimation (*M* = −0.010, *SE* = 0.007), *F*(1,51) = 7.516, *p* = 0.008, *η_p_*^2^ = 0.128. Irrespective of the direction of the error, it is consistent with the findings of Experiment 2, as detailed in the main text.

**Table A1:** Stimulus material used in Experiment 1.

Decision Problem	Risky Option	Certain Option
1	10, 0.1	2, 1
2	5, 0.1	0.5, 1
3	10, 0.2	2, 1
4	5, 0.2	1, 1
5	10, 0.25	2.5, 1
6	5, 0.25	0.75, 1

Note. For each option, only one outcome was given, followed by its probability; the second outcome, which is not stated, was 0 and occurred with a probability complementary to the stated one. For instance, the outcomes of the risky option in Problem 1 were CNY 10 with a probability of 0.1 and CNY 0 with a probability of 0.9; the outcomes of the certain option in Problem 1 were CNY 2 with a probability of 1.

**Table A2:** Stimulus material used in Experiment 2.

Decision Problem	Risky Option	Certain Option
1	10, 0.4	4, 1
2	5, 0.4	2, 1
3	10, 0.5	5, 1
4	5, 0.5	2.5, 1
5	10, 0.6	6, 1
6	5, 0.6	3, 1
7	10, 0.8	8, 1
8	5, 0.8	4, 1
9	10, 0.9	9, 1
10	5, 0.9	4.5, 1
11	10, 0.75	7.5, 1
12	5, 0.75	3.75, 1

Note. Table A2 is interpreted in the same way as Table A1.
